# Advances in CT features and radiomics of checkpoint inhibitor-related pneumonitis: A short review

**DOI:** 10.3389/fimmu.2023.1082980

**Published:** 2023-01-23

**Authors:** Jie Huang, Xueqin Chen, Bing Xia, Shenglin Ma

**Affiliations:** Department of Thoracic Oncology, Affiliated Hangzhou Cancer Hospital, Zhejiang University School of Medicine, Hangzhou, China

**Keywords:** checkpoint inhibitor-related pneumonitis (CIP), computed tomography (CT), radiomics, radiation-induced pneumonitis (RIP), cancer

## Abstract

Checkpoint inhibitor-related pneumonitis (CIP) is a complication of immunotherapy for malignant tumors that severely limits the treatment cycles as well as endangers patients’ health. The chest CT imaging features or typing of CIP and the application of radiomics will contribute to the precise prevention, early diagnosis and instant treatment of CIP. This article reviews the advances in the CT features and the application of radiomics in CIP.

## Introduction

Programmed death 1 (PD1)/programmed death-ligand 1 (PD-L1) targeted immune checkpoint inhibitors (ICIs) have revolutionized cancer treatment. With the continuous publication of results from large randomized controlled clinical studies, ICIs alone or in combination with radiotherapy, chemotherapy, or anti-angiogenic therapy are becoming the first-line treatment of choice for most major cancer types (1–4). However, the incidence of immunotherapy-related adverse effects (irAEs) is inevitable and relatively high. For example, a meta-analysis with a total of 1063 Chinese patients in 13 clinical studies enrolled, of whom 922 (86.7%) received ICIs monotherapy and 141 (13.3%) received ICIs plus chemotherapy or anti-angiogenesis, reported that the incidence of irAEs of any grade was 43.3%, and 4.3% of patients discontinued the treatment due to the severe irAEs (5). The incidence of checkpoint inhibitor-related pneumonitis (CIP) was reported to range from 1%-4% with single-agent immunotherapy (6) and up to 6.6% with combined strategies (7). Early clinical symptoms are not easy to detect, and severe grade 3-5 CIP may lead to severe respiratory failure and is one of the major fatal adverse reactions, so it is urgent to identify or predict the occurrence of immunotherapy-related pneumonitis accurately.

CIP can be diagnosed by meeting the following three criteria: (i) history of ICI medication; (ii) newly appeared lung shadow; (iii) excluding lung infection, lung tumor progression, other causes of interstitial lung disease, pulmonary embolism, pulmonary vasculitis and pulmonary edema. However, during the treatment of tumor patients, other factors such as infections, radiation therapy, other drugs such as targeted drugs, chemotherapy, etc. are often mixed, thus it is often difficult to accurately diagnose CIP and select the appropriate treatment plan accordingly timely. Although some biomarkers such as interleukin (IL) including IL2 and IL17a, as well as circulating CD8+ T-cells and neutrophil/lymphocyte ratio have been found to correlate with the incidence of CIP (8). It has also been suggested that chronic lung disease such as chronic obstructive pulmonary disease, emphysema, and interstitial lung disease and history of prior chest radiation therapy may be independent risk factors for the occurrence of CIP, but there are no clear, stable, and mature identification or prediction models that can be applied in the clinical setting.

Radiomics is an emerging technology in medical imaging with the automated extraction of multidimensional imaging data from medical images aiming for comprehensive visualization and characterization of the disease-involved tissue and its microenvironment. Radiomics-based approaches that quantitatively identify associations between the extracted imaging data and clinical characteristics or outcomes and use these associations to construct predictive models thereby providing a solution to clinical problems, such as those that have been developed for identification of benign or malignant small pulmonary nodules, interpretation of COVID-19 pneumonia, and other various aspects of the medical field. While there are many studies on the use of radiomics to predict the efficacy of immunotherapy, there is no systematic review of the emerging field of radiomics to identify or predict the occurrence of CIP, although several studies were published in recent years. Therefore, we attempt to review the relevant content and provide some insights.

## CT imaging patterns of CIP

Computed tomography (CT) is an important imaging modality for the diagnosis of lung disease, and the imaging features on CT of the lung are crucial to diagnose CIP correctly. American Thoracic Society/European Respiratory Society (ATS/ERS) revised and supplemented the international consensus on the classification and diagnostic criteria of interstitial pneumonia in 2013 (9), and subsequent scholars have mostly based their phenotypic patterns of CIP images on these criteria, which include organizing pneumonia (OP), nonspecific interstitial pneumonia (NSIP), hypersensitivity pneumonitis (HP), bronchiectasis, and acute interstitial pneumonia - acute respiratory distress syndrome (AIP-ARDS).

In 2016, Naidoo et al. first classified the following imaging subtypes based on CT in 27 patients with CIP: cryptogenic organizing pneumonia (COP) (2/27, 19%), ground glass opacity (GGO) (10/27, 37%), NSIP (2/27, 7%) and HP (6/27, 22%), and not otherwise specified (NOS) pneumonia (4/27, 15%), and found that CT imaging patterns were essentially consistent throughout the patients’ clinical course, except for two patients (10). Lin et al. found that predominant patterns were GGO (43.6%), NSIP (25.5%), COP (18.2%), and follower by NOS (12.7%) based on a similar classification pattern, and AIP-ARDS indicated severe pneumonia (correlation coefficient = 0.707, *p* < 0.001) (11). However, the above study analyzed the radiographic element GGO together with the imaging patterns, which was not appropriate. According to the diagnostic criteria for interstitial pneumonia published by ATS/ERS, Delaunay et al. instead analyzed in a larger sample size (64 cases) and found COP to be the most common pattern (15/64, 23.4%), followed by HP (10/64, 15.6%), NSIP and bronchiectasis in 7.8% (5/64) and 6.3% (4/64), respectively and another 23 with unspecified pneumonia (35.9%) (12). Subsequent published studies consistently found the COP pattern as the most common type in both lung and non-lung tumors (13–15). There was no significant difference in imaging patterns between early-onset and late-onset CIP (15). The AIP/ARDS type turned out to have the highest adverse effect grade, while NSIP and HP seemed to be mild (median grade: 3, 2, 1, 1; *p* = 0.006) (13).

Specifically looking at the CT radiographic elements, GGO was the most common feature (52/64, 81.3%), followed by consolidation (34/64, 53.1%), bronchiectasis (11/64, 17.2%), interlobular septal thickening (10/64, 15.6%) and intralobular lines (14/64, 21.9%), etc. and mainly showed diffuse lung involvement (12). Consolidation was common in patients with lung cancer and less frequent in non-lung cancer patients (29%), while nodular lesions were found in only a minority of patients in the non-lung cancer group (29%) (14). Interestingly, Balaji et al. specifically looked at the steroid-refractory CIPs, and reported a similar pattern, with GGOs (50%, 6/12) as the predominant ones and mostly involving bilateral lung fields (75%, 9/12) (16). Similarly, Imran et al. reported a few cases of rapid deterioration even after adequate treatment with CT features of diffuse GGOs (17), however, the imaging features are similar in common CIPs without novel findings, which should be further explored and well organized.

## Identification of CIP by CT radiomics

Radiation therapy is often involved and plays an important role in the immunotherapy process, improving local control of the tumor as well as acting as an immune adjuvant to sensitize the efficacy of immunotherapy. However, in the case of non-infectious pneumonia, the attribution of pneumonia by radiation or immunotherapy is often difficult for clinicians to distinguish, thereby making it difficult to treat. Hence, using radiomics to analyze the differences in imaging features between radiation induced pneumonitis (RIP) and CIP and to classify and identify them will help clinical decision making and benefit patients.

The first systematic comparison of CT features of RIP and CIP was performed by Chen et al. at Johns Hopkins University, which included 82 patients: 30 after RT+ICI, 29 after thoracic RT, and 23 after ICI. Compared with RIP, CIP was more likely to be bilateral (65% vs. 28%; p = 0.01), involve more lobes (66% vs. 45%), and was less likely to have sharp borders (17% vs. 59%; p = 0.004). The area under curve (AUC) of the machine learning model to differentiate CIP and RIP reached 0.76 based on the following 7 imaging features: bilateral, number of lobes, volume of lung involved, multifocal, radiographic elements, radiographic patterns, and sharp border (18). A similar radiomics study done by Cheng et al. in China, developed the linear SVM classification model based on three radiomics features (intensity histogram, bag-of-words [BoW] features, and gray-level co-occurrence matrix [GLCM]), and a 10-fold cross-validation in patients receiving only ICI or RT showed robust results with AUCs of 0.937. The model was then tested in patients receiving ICI+RT and could achieve an AUC of 0.896 (19). The retrospective study by Qiu et al. included a larger sample (126 cases) and finally identified the Rad-score (11 imaging histological features) with the potential to distinguish between CIP and RIP, and also found that bilateral involvement and sharp border were associated with the distinguishment of CIP and RIP. Combining the Rad-score and the above two features, authors created a robust model showing good performance in both the training dataset (empirical-based AUCs of 0.953) and the validation dataset (AUC = 0.947) (20), which is also the model with the best recognition performance reported in the literature so far.

These results suggest that CT-based radiomics has good potential in differentiating CIP from RIP in lung cancer and may become a practical tool in the future to provide a valuable differential diagnosis for the attribution of pneumonia in patients treated with concomitant ICI and RT.

## Prediction of CIP by CT radiomics

Treatment after the onset of CIP is often difficult to ensure patients’ quality of life and long progression-free survival because of the severity of the disease, making it a hot issue to predict the possible onset of CIP well before ICI treatment and to perform primary prevention or to screen out the optimal population. The earliest exploration was conducted by Colen et al. at MD Anderson, who analyzed a total of 1860 imaging features on baseline chest CT from 2 patients who developed CIP and 30 patients who did not develop CIP. Using feature selection methods of maximum correlation and minimum redundancy, abnormality detection algorithms, and leave-out cross-validation, 2 radiological features with significant differences were finally identified: skewness (a measure of histogram symmetry) and angular variance of the sum of squares (a measure of dispersion) (21). Spiele et al. from the University of Miami, on the other hand, performed a proof of concept for the prediction of pneumonia after radiation therapy combined with immunotherapy in a mouse Lewis lung cancer model. Mice were bilaterally imaged with CT and MRI after subcutaneous tumor formation and blood collection, and then treated with RT of the right abdominal tumor only (3*8Gy) followed by intraperitoneal injection of PD-1 inhibitors. They found that 3 CT radiomic features (mean grayscale, histogram kurtosis and co-occurrence matrix entropy) and 1 MRI feature (histogram kurtosis) together with baseline neutrophil-to-lymphocyte ratio (NLR) and granulocyte-macrophage colony-stimulating factor (GMSF) levels were positively correlated with CD45 infiltration (22). However, it is important to note that this model only assessed the CD45 infiltration levels to indicate the occurrence of pneumonia is not sufficiently reasonable and could not distinguish between RIP and CIP. Recently, Tan et al. retrospectively collected baseline CT images and clinical data from 24 patients who experienced CIP after immunotherapy and 24 controls who did not experience CIP. The model was pre-trained using a two-stage migration learning on a large natural image dataset and a large CT image dataset of pneumonia, then finally trained on locally collected CT image data. Finally, contrast learning was used to mine high-performance imaging feature models. Using five-fold cross-validation, the model was able to accurately predict CIP patients and non-ICIP patients with an AUC of 0.918 and an accuracy of 0.920 (23). This study strongly indicates that deep learning has great potential for identifying patients at risk of developing CIP.

From these studies, we could conclude that the prediction models proposed by big data-based radiomics studies may lead to effective risk stratification, close monitoring and timely management of CIP in the future to improve treatment outcomes. However, given the complexed clinical course of CIP, above radiomics studies did not show the exact prediction ability of those >3 grade CIPs or those rapid deteriorating CIPs that warrant emergent treatments.

## Shortcomings and challenges of radiomics to predict or identify CIP

The application of radiomics in CIP has attracted some scientific interest, and in general, some excellent findings on the prediction and identification of CIP by radiomics have been reported. However, it has to be pointed out that there are still many problems from the mature development of the model and its real application in the clinic: (1) the image input, including the technical factors of CT imaging and the segmentation of region of interest (ROI). Although chest CT has been widely used in major hospitals, differences in hardware, scanning protocols and reconstruction algorithms of different manufacturers still have an impact on the extraction of image histological features. Secondly, the segmentation of ROI has been studied either artificially by experts or using software segmentation, but the repeatability of segmentation among different segmentation experts or even within software still needs to be improved (24, 25). (2) In terms of model maturation and validation, most of the current radiomics studies have small sample sizes, and more prospective studies with larger sample sizes are needed in the future to build models that can uncover more valuable radiomics features, and indeed more external data are needed to validate the accuracy of radiomics models. In addition, it is worth looking forward to whether the combination of radiomics with other available data such as pathology, genomic alterations or a variety of blood test results can bring more robustness and accuracy to the model. (3) As for the molecular biological significance of the model, for example, most of the radiomics features identified in the prediction study are pre-defined artificial features, and the potential molecular biological significance of these features needs to be further explored. However, because the mechanisms of CIP occurrence are still largely unclear, it is difficult to conduct relevant studies. In addition, radiomics studies of paired pre- and post-occurrence of CIP are rarely reported, and only correlational findings are shown, thus it is worthwhile to analyze whether causal findings can further improve the predictive performance. (4) For the prediction or identification of severe CIPs that need emergent treatment, and prediction of steroid-refractory or rapid progressing ones without relief modalities, still no radiomics data are available and relevant studies are thereby recommended.

## Author contributions

JH conceived the concept, created the draft. XC, BX and SM discussed and revised the manuscript. All authors contributed to the article and approved the submitted version.
